# Supplementary material for: Cost-effectiveness of virtual emergency care models: A protocol for a systematic review
Source: PLoS One. 2025 Sep 4;20(9):e0330946. doi: 10.1371/journal.pone.0330946 (PMC12410708; doi:10.1371/journal.pone.0330946)
Supplement: S1 File — (DOCX) [file pone.0330946.s002.docx]

**Appendices**

**Appendix 1: Data extraction form**

| **S.No** | **Category** | **Variable** |
| --- | --- | --- |
|  | Bibliographic Details | First author |
|  |  | Publication year |
|  |  | Journal |
|  |  | Funding sources |
|  | Participants | Inclusion/exclusion criteria |
|  |  | Sample size |
|  |  | Age (mean, SD, range) |
|  |  | Sex (% female) |
|  |  | Emergency condition(s) |
|  |  | Triage acuity level |
|  |  | Comorbidities |
|  | Interventions | Virtual care model (e.g. ED-based telemedicine, teletriage, remote monitoring) |
|  |  | Technological medium (e.g. video, telephone, web, mobile app) |
|  |  | Provider type (e.g. physician, nurse, advanced practice provider) |
|  |  | Frequency and duration |
|  |  | Relationship to in-person care (e.g. standalone, triage, step-up/step-down) |
|  | Comparators | Description of usual in-person emergency care |
|  |  | Providers, processes, and resources involved |
|  | Outcomes | Incremental cost-effectiveness ratio (ICER) and components |
|  |  | Type and source of effectiveness data (e.g. QALYs from trial, ED LOS from admin data) |
|  |  | Type and source of cost data (e.g. payer, hospital, patient costs) |
|  |  | Analysis perspective (e.g. healthcare system, societal) |
|  |  | Time horizon |
|  |  | Discount rate |
|  |  | Sensitivity analyses (e.g. probabilistic, deterministic) |
|  |  | Subgroup analyses |
|  | Study Details | Country and setting |
|  |  | Analytic approach (e.g. within-trial, model-based) |
|  |  | Model structure and assumptions |
|  |  | Utility scores and sources |
|  |  | Cost sources and currency |
|  |  | Missing data approach |
