# Supplementary material for: Cost-effectiveness of virtual emergency care models: A protocol for a systematic review
Source: PLoS One. 2025 Sep 4;20(9):e0330946. doi: 10.1371/journal.pone.0330946 (PMC12410708; doi:10.1371/journal.pone.0330946)
Supplement: Attachment — Submitted filename: Response to Reviewers.docx [file pone.0330946.s004.docx]

**Response to Reviewer Comments and Manuscript Changes**

We thank both the Editor and reviewer for their thoughtful and constructive feedback on our manuscript. We have carefully addressed each point and made appropriate revisions to strengthen the protocol. Below we provide detailed responses to each comment.

**Response to Editor Comments**

| **S.No.** | **Editor Comment** | **Response** | **Changes Made in Manuscript** |
| --- | --- | --- | --- |
| 1 | Please move the planned use of the PICOS framework, the use of the PRISMA guidelines and the CHEERS checklist to the appropriate place in the Methods section. | We thank the editor for this important structural suggestion. We have moved the description of PICOS, PRISMA, and CHEERS from the separate "Frameworks" section to their appropriate locations within the Methods section for better organization and flow. | **Deleted:** Entire "Frameworks" section (lines 83-102 in original manuscript)  **Added:** After "Methods" heading (line 92): "This systematic review will be conducted and reported according to the Preferred Reporting Items for Systematic review and Meta-Analysis (PRISMA) guidelines [33]" (lines 93-94)  **Added:** After "Eligibility criteria" heading (line 95): "We will select studies according to the following PICOS (Population, Intervention, Comparator, Outcomes, Study design) criteria:" (lines 96-97)  **Added:** Additional text about preliminary scoping search and implementation barriers (lines 81-90) |
| 2 | CHEERS is a reporting checklist for health economic evaluations, the Drummond checklist is to assess the quality of economic evaluation, and the CHEC checklist is a tool to assess the methodological quality of economic evaluation. These are used interchangeably in the manuscript as a checklist, quality assessment tool and guideline for economic evaluation. Please correct this throughout the manuscript and make appropriate adjustments. | We acknowledge this important clarification and have corrected the terminology throughout the manuscript. CHEERS is now consistently referred to as "reporting standards" for data extraction, while Drummond and CHEC are referred to as "quality assessment tools" for evaluating methodological quality and risk of bias. | **Line 32:** Changed "assess methodological quality and risk of bias using the Drummond and CHEC checklists" to "assess methodological quality and risk of bias using established quality assessment tools"  **Line 181:** Changed "A standardized data extraction form will be developed based on the CHEERS checklist" to "A standardized data extraction form will be developed based on the CHEERS reporting standards"  **Lines 209-211:** Changed "using the Drummond 10-item checklist and the Consensus Health Economic Criteria (CHEC) 19-item checklist" to "using the Drummond 10-item quality assessment tool and the Consensus Health Economic Criteria (CHEC) 19-item quality assessment tool"  **Line 214:** Changed "The CHEC checklist assesses" to "The CHEC tool assesses"  **Line 296:** Changed "adapted from the CHEERS checklist" to "adapted from the CHEERS reporting standards"  **Lines 324-325:** Changed "established economic evaluation guidelines (CHEERS, Drummond, CHEC)" to "established economic evaluation reporting standards (CHEERS) and quality assessment tools (Drummond, CHEC)"  **Added:** New sections on "Stakeholder engagement" (lines 265-270) and "Living systematic review approach" (lines 271-276) |

**Response to Comments from Reviewer 1**

| **S.No.** | **Reviewer Comment** | **Response** | **Changes Made in Manuscript** |
| --- | --- | --- | --- |
| 1 | Data policy/sharing: I think it is necessary to include the statement "No datasets were generated or analysed during the current study. All relevant data from this study will be made available upon study completion." within the manuscript text. I suggest at the end of methods, or elsewhere as suggested by the editors. If included in methods, it could be combined with further details on how a living systematic review will be maintained. | We agree and have added a comprehensive data availability statement that also addresses the living systematic review approach. | Added separate sections for "Stakeholder engagement" (lines 265-270), "Living systematic review approach" (lines 271-276), and formal "Data availability" statement (lines 401-403). |
| 2 | Ln 28 & 54: "from inception to February 2025" - elsewhere it states that publications prior to 2010 will be exluded. Amend or explain further why including the in the search. | We acknowledge this inconsistency. We have corrected the search timeframe to align with our exclusion criteria. | Line 28: Changed "from inception to February 2025" to "from 2010 to February 2025"  Line 149: Changed "from their inception to February 2025" to "from 2010 to February 2025" |
| 3 | Ln 46: Statistic is unclear. Is it 4.4% of total US health expenditure? | Yes, this refers to total US health expenditure. We have clarified this for precision. | Line 47: Changed "In 2017, US EDs accounted for 4.4% of total health expenditures" to "In 2017, US EDs accounted for 4.4% of total US health expenditures" |
| 4 | Ln 61: This statistic is unclear. should it say "increased 16-fold between January and July 2020 compared with the same time period in 2019"? | Correct, we have clarified the time period comparison for better precision. | Line 62: Changed "ED telemedicine visits increased 16-fold from January to July 2020 compared to 2019" to "ED telemedicine visits increased 16-fold between January and July 2020 compared with the same time period in 2019" |
| 5 | Ln 131-132: In outcomes, make it clear that you will include reported costs and effects even if these are not reported with an ICER. | We agree this clarification is important and have modified the outcomes section accordingly. | Lines 126-128: Changed "Secondary outcomes include incremental costs and effects, net monetary benefit, and sensitivity analyses" to "Secondary outcomes include incremental costs and effects (even when not reported as ICERs), net monetary benefit, and sensitivity analyses" |
| 6 | Inclusions/Exclusions: make it clearer what countries will be included in the review and what languages (all?) | We have added explicit geographic and language scope information to address this important methodological detail. | Added after line 141: "**Geographic and language scope:** Studies from all countries will be included to capture the global evidence base on virtual emergency care cost-effectiveness. No language restrictions will be applied, ensuring that relevant studies published in any language are considered for inclusion. Efforts will be made to translate non-English studies where necessary to facilitate comprehensive analysis." (lines 142-146) |
| 7 | Ln 247: add reference at end of this line for methods described. | We have added an appropriate reference for meta-analysis methods. | Line 242: Added reference [43] after "meta-analysis" and added "Borenstein, M., et al., Introduction to meta-analysis. 2021: John wiley & sons." to references (reference 43) |
| 8 | Ln 248: I believe "2" of "I2" should be super-script. | Correct, we have fixed the formatting. | Line 243: Changed "I2" to "I²" |
| 9 | Ln 251: Add reference at the end of the line. | We have added the appropriate reference for Egger's test. | Line 246: Added reference [44] and added "Egger, M., et al., Bias in meta-analysis detected by a simple, graphical test. Bmj, 1997. 315(7109): p. 629-34." to references (reference 44) |
| 10 | Ln 353-354: making this a living systematic review is commendable. I think some further description should be added to the methods. Also, please include reference (Cochrane 2019). | We appreciate this feedback and have expanded the living systematic review methodology in the methods section. | Added section "Living systematic review approach" (lines 271-276): "This systematic review will be maintained as a living review, with planned updates conducted annually or when substantial new evidence becomes available. Updated searches will use the same methodology, with screening and data extraction conducted by the same research team to ensure consistency. Stakeholders will be notified of significant updates through our knowledge translation networks [46]." Added Elliott reference (reference 46). |
| 11 | Ln 363: Include reference for "quadruple aim". Berwick 2018 & Sikka BMJ 2015? | We have added the appropriate references for the quadruple aim concept. | Line 361: Added references [48, 49] and added both Berwick and Sikka references to reference list (references 48 and 49) |
| 12 | Ln24-26: Replace with objective given in main text as I found this clearer. | We agree the main text objective is clearer and have updated the abstract accordingly. | Lines 24-26: Changed to "This systematic review aims to evaluate the cost-effectiveness of virtual emergency care models compared to in-person emergency care." |
| 13 | Ln 29: suggest after "evaluation" add "that report both costs and effects" | This clarification improves precision about our inclusion criteria. | Line 29: Changed to "for economic evaluations that report both costs and effects comparing virtual and in-person emergency care models" |
| 14 | Ln 29: Add exclusions: "Studies that compare multiple virtual interventions without an in person care comparator will be excluded" as advised by PRISMA | We have added this exclusion criterion to the abstract as suggested. | Added lines 30-31: "Studies that compare multiple virtual interventions without an in-person care comparator will be excluded." |
| 15 | Ln 64-65: Will these barriers be explored in the review? If so, this could be made clearer in methods/discussion. | While not our primary focus, we will extract barrier information when available. We have clarified this. | Added after line 66: "While our primary focus is on economic outcomes, we will extract and report implementation barriers and facilitators as secondary findings when reported in included economic evaluations." Also clarified in objective section (lines 87-90). |
| 16 | General: has a scoping search been done and, if so, what were the findings? | We conducted a preliminary scoping search and have added this information. | Added after line 80: "A preliminary scoping search conducted in PubMed identified approximately 150 potentially relevant studies, suggesting sufficient literature exists to warrant this comprehensive systematic review. The scoping search revealed heterogeneity in virtual care modalities, outcome measures, and economic evaluation methods, supporting our planned narrative synthesis approach." (lines 81-85) |
| 17 | Ln 84-103: I find this "Frameworks" section a helpful summary. Perhaps add CHEC/Drummond, SWiM and GRADE. | We agree this would make the frameworks section more comprehensive. | The frameworks mentioned are now integrated throughout the methods section where they are specifically applied: CHEC/Drummond in Risk of bias assessment section (lines 207-218), SWiM in Synthesis section (lines 224-225), GRADE in Confidence in cumulative evidence section (lines 253-256) |
| 18 | Ln 125-126: Delete final sentence as this is covered in "exclusions". | We agree this is redundant and have removed it. | Deleted redundant sentence from intervention section: "We will exclude studies comparing multiple virtual modalities without an in-person ED care arm." |
| 19 | Ln 143-144: Delete final sentence as this is covered in "exclusions". | We agree this is redundant and have removed it. | Deleted redundant sentence about partial economic evaluations from trial-based evaluations section (originally in lines 143-144) |
| 20 | Ln 243-245: Can you provide a reference for this? Example of where it's been done before? | We have added appropriate references for outcome standardization methods. | Added after line 240: "following established methods for standardizing economic outcomes across studies [41, 42]" and added corresponding references (Petrillo and Drummond references) |
| 21 | Ln 279: replace "and" with ", which". | Corrected for better grammar flow. | Line 286: Changed "and is a key strength" to "which is a key strength" |
| 22 | Ln 286: replace "was" with "will be". | Corrected verb tense for consistency. | Line 293: Changed "was iteratively developed" to "will be iteratively developed" |
| 23 | Ln 290-293: Long and confusing sentence. Suggest full stop after "studies" on ln 292, then rewording remaining part of sentence. | We have restructured this for better clarity. | Lines 297-300: Split into two sentences: "Assessment of methodological quality and risk of bias using the Drummond and CHEC checklists will provide a transparent evaluation of the internal and external validity of included studies. Results will inform the certainty of evidence ratings using the GRADE approach." |
| 24 | Ln 295: if possible, add reference by way of an example of pooled ICERs. | We have added example references of ICER pooling in economic evaluations. | Added after line 302: "as demonstrated in previous systematic reviews such as Noparatayaporn et al. [47], which evaluated the cost-effectiveness of bariatric surgery across different patient subgroups and time horizons through a meta-analysis of incremental net monetary benefit." |
| 25 | Ln 299-311: Whole paragraph is repetitive of introduction and I think could be removed here. | We agree this paragraph is redundant and have removed it. | Removed the repetitive paragraph about the protocol being timely and relevant (the content was streamlined and integrated elsewhere in the discussion) |
| 26 | Ln 339: add "s" at end of "system" | Corrected for proper grammar. | Line 337: Changed "health system" to "health systems" |
| 27 | Ln 348-350: I would consider involving representitives at an earlier stage in your reserach (as early as possible) and describing their inolvement, how this can inform the research, in the methods. | Excellent suggestion. We have added early stakeholder engagement to the methods section. | Added "Stakeholder engagement" section (lines 265-270): "Representatives from key stakeholder groups (emergency physicians, health economists, hospital administrators, payers, and patient advocates) will be engaged early in the review process to inform data extraction priorities, outcome interpretation, and knowledge translation planning. This engagement will occur through structured consultations during protocol refinement and preliminary findings review." |

We believe these revisions have substantially strengthened the protocol while maintaining its core methodological rigor. The added details will enhance reproducibility and implementation. We welcome any additional feedback from the reviewers.
