# Supplementary material for: Cost-effectiveness of virtual emergency care models: A protocol for a systematic review
Source: PLoS One. 2025 Sep 4;20(9):e0330946. doi: 10.1371/journal.pone.0330946 (PMC12410708; doi:10.1371/journal.pone.0330946)
Supplement: Attachment — Submitted filename: Response_to_Reviewers_auresp_2.docx [file pone.0330946.s005.docx]

**Response to Editor Comments and Manuscript Changes**

We thank the Editor for the thoughtful and constructive feedback on our manuscript. We have carefully addressed each point and made appropriate revisions to strengthen the protocol. Below we provide detailed responses to each comment.

| **S.No.** | **Editor Comment** | **Response** | **Changes Made in Manuscript** |
| --- | --- | --- | --- |
| 1 | **Journal Requirements:** If the reviewer comments include a recommendation to cite specific previously published works, please review and evaluate these publications to determine whether they are relevant and should be cited. There is no requirement to cite these works unless the editor has indicated otherwise. | We acknowledge this requirement. We have reviewed all citations for relevance to our systematic review protocol on virtual emergency care cost-effectiveness. All included references are directly relevant to emergency medicine, telemedicine, health economics, or systematic review methodology. | **Completed.** All references have been reviewed and confirmed as relevant to the study objectives. No additional citations were deemed necessary. |
| 2 | **Reference List Review:** Please review your reference list to ensure that it is complete and correct. If you have cited papers that have been retracted, please include the rationale for doing so in the manuscript text, or remove these references and replace them with relevant current references. Any changes to the reference list should be mentioned in the rebuttal letter that accompanies your revised manuscript. | We have completed a comprehensive review of our reference list. All 49 references have been verified as current, accurate, and not retracted. All references are appropriate for the topics covered (emergency medicine, telemedicine, health economics, and systematic review methodology). | **Completed.** Reference list has been thoroughly reviewed and verified. All references are current and appropriate. No retracted papers were identified. |
| 3 | **Specific Reference Correction:** Please recheck the references cited in the manuscript. Update the reference cited for Dilokthornsakul et al. [47] as the paper is not related to seasonal influenza vaccination. | We have corrected this error. The reference [47] has been updated to accurately reflect Noparatayaporn et al.'s work on bariatric surgery cost-effectiveness, which is the appropriate citation for the context in which it appears in our manuscript. The previous incorrect reference to seasonal influenza vaccination has been removed. | **Completed.**  - **Lines 303-305:** Removed incorrect reference to "Dilokthornsakul et al. [47] on seasonal influenza vaccination"  - **Lines 305-306:** Corrected text to properly reference "Noparatayaporn et al. [47] on bariatric surgery cost-effectiveness meta-analysis"  - **Reference [47]:** Confirmed as "Noparatayaporn, P., et al., Incremental Net Monetary Benefit of Bariatric Surgery: Systematic Review and Meta-Analysis of Cost-Effectiveness Evidences. Obesity Surgery, 2021. 31(7): p. 3279-3290." |
